# Choosing an Effective Compatibilizer for a Virgin HDPE Rich-HDPE/PP Model Blend

**DOI:** 10.3390/polym13203567

**Published:** 2021-10-16

**Authors:** Erdal Karaagac, Thomas Koch, Vasiliki-Maria Archodoulaki

**Affiliations:** Institute of Materials Science and Technology, Faculty of Mechanical and Industrial Engineering, TU Wien, Getreidemarkt 9, 1060 Vienna, Austria; thomas.koch@tuwien.ac.at (T.K.); vasiliki-maria.archodoulaki@tuwien.ac.at (V.-M.A.)

**Keywords:** high density polyethylene, polypropylene, compatibilization, recycling

## Abstract

The most widely used commodity polymers in the rigid packaging industry are polypropylene (PP) and high-density polyethylene (HDPE). For example, blow molding grade of HDPE as a bottle and injection molding grade of PP as a cap are often used to produce detergent bottles. Therefore, the recycled HDPE bottles from post-consumer waste include PP as a contaminant originated from PP bottle caps. To simulate mechanical recycling of bottle waste, the mechanical properties of HDPE-rich-HDPE/PP virgin model blend were studied. For compatibilization, ethylene-based olefin block copolymer, propylene-based olefin block copolymer, ethylene propylene random copolymer, and styrene-butadiene-styrene triblock copolymer were chosen as potential compatibilizer candidates. Contact angle measurements, morphological analysis, adhesion tests of compatibilizer candidates to polymer blend components and the tensile as well as tensile impact properties of the ternary blends were studied. It was found that the ethylene-based olefin block copolymer was the most effective compatibilizer resulting in a return of mechanical properties to those of neat vHDPE due to its ability to encapsulate dispersed vPP particles in a vHDPE matrix (core-shell morphology) and the best adhesion to polymer blend components.

## 1. Introduction

Polypropylene (PP) and polyethylene (PE) are the most abundant thermoplastic resins with a share of 49.2% of European plastic demand in 2019 [1]. The polypropylene (PP) and high-density polyethylene (HDPE) are widely used in the rigid packaging industry. One example of such an application of high-density polyethylene (HDPE) as well as polypropylene (PP) in rigid packaging industry is the production of detergent bottles. Such bottles are produced by extrusion blow molding process consisting of blow molding grade of high-density polyethylene (HDPE) as a bottle and injection molding grade of polypropylene (PP) as a cap or closure on the bottle. Separation of individual polymer types during mechanical recycling is difficult due to the similar densities of polypropylene (PP) and high-density polyethylene (HDPE). Therefore, the HDPE post-consumer waste from blow-molding applications most likely contains contaminants from PP. However, the presence of PP as a contaminant in PE results in poor mechanical properties, especially in impact resistance due to immiscibility of polymer blend components (PP and PE), high interfacial tension, phase separated morphology and the lack of interfacial adhesion [2,3,4,5,6,7].

A method for the improvement of interfacial adhesion between two phases and thereof mechanical properties of immiscible blends by the addition of proper pre-made block or random copolymer is known as compatibilization [8]. This pre-made block or random copolymer as a compatibilizer has a molecular architecture with miscible chains to both polymer blend components, which adhere two polymers together at the interface boundary like a bridge with interfacial entanglements. The requirements for successful compatibilization are the encapsulation of dispersed phase by compatibilizer (core-shell morphology) and sufficient adhesion of compatibilizer to polymer blend components for better stress transfer at the interface boundary [9]. Generally, successful compatibilization can be recognized by improvements in impact resistance against dynamic as well as continuously applied loads [10].

The addition of compatibilizer to a binary blend results in a ternary blend. The morphology determines final mechanical properties. According to the literature, ternary blends morphology can be estimated using spreading coefficients calculated by interfacial tensions [11,12,13,14]. Zhang et.al [12] described the hierarchical factors in order of priorities, such as interfacial adhesion, interfacial tension, viscosity ratio and shear stress that affect the final phase morphology of ternary blend. Interfacial adhesion of compatibilizer to polymer blend components accomplished by interfacial interactions, such as interfacial entanglements or co-crystallization with the chains of polymer blend components upon cooling depend on molecular weight of block copolymer [15,16,17,18,19], side chain length of graft copolymer [20,21], the compositional distribution of block copolymer in terms chemical architecture [22,23] and tie layer thickness [22,23,24]. Hemmati et al. [13] investigated the influence of melt viscosity and interfacial interaction on morphology of ternary blends. They found that the encapsulation of dispersed phase by compatibilizer (core-shell morphology) was controlled by interfacial tensions rather than melt viscosity ratios of polymer components.

According to the literature, a typical PP contamination range in HDPE is 3–12 wt% for bottle waste stream from blow molding applications [25]. Therefore, the recycled post-consumer bottle waste by melt blending is in fact a PE-rich PE/PP blend with poor impact resistance. In order to evaluate the compatibilization effect of a compatibilizer, model virgin blends are mostly used. The compatibilization of virgin PP/PE blends with various type of compatibilizers, such as ethylene propylene elastomer (EPR), ethylene propylene diene copolymer (EPDM), styrenic block copolymer (SBC), ethylene-octene copolymer (EOC) and olefin block copolymer (OBC), has been reported [10]. The ethylene segment of OBC is miscible with polyethylene, whereas the octene segment of OBC is miscible with polypropylene [26]. The ethylene segment and propylene segments of EPR are miscible with polyethylene and polypropylene, respectively [5]. SEBS makes PP/PE blend compatible through their butylene and ethylene segments [27]. The miscible segments in molecular architecture of compatibilizers with polymer blend components enable interfacial entanglements and better adhesion. For this purpose, olefin block copolymer (OBC), ethylene-propylene random copolymer (EPR) and styrene-ethylene-butylene-styrene triblock copolymer (SEBS) are chosen as compatibilizer candidates due to their miscible segments in polyethylene and polypropylene. In fact, selection of the most effective compatibilizer from various types of compatibilizers for the compatibilization of virgin blow molding grade high density polyethylene (vHDPE)-rich/injection molding grade polypropylene (vPP) remains an open challenge. This work focuses on compatibilization of a virgin model blend of 10 wt% injection molding grade PP (vPP) and 90 wt% blow molding grade HDPE (vHDPE) as a very common composition to simulate recycling of post-consumer detergent bottle waste.

In order to determine the most effective compatibilizer for the compatibilization of 10 wt% vPP (injection molding grade) contaminated vHDPE (blow molding grade) model virgin blend (HDPE-rich-HDPE/PP blend), a set of methods based on estimation of morphology from contact angle measurements, morphological analysis, adhesion tests of compatibilizer candidates to polymer blend components and tensile as well as tensile impact properties of ternary blends with compatibilizer candidates have been applied.

## 2. Materials and Methods

### 2.1. Materials

Blow molding grade of HDPE homopolymer (Hostalen GF 4750) with a melt flow rate (MFR) of 0.4 g/10 min (190 °C/2.16 kg) was purchased from Lyondellbasell. Injection molding grade of isotactic PP (HF 7005A) with a melt flow rate (MFR) of 21 g/10 min (240 °C/2.16 kg) was purchased from Borealis. The HDPE and PP were used for binary blend preparation. For the compatibilization of binary HDPE rich HDPE/PP blend, various types of compatibilizer candidates were used. All used materials and compatibilizer candidates are listed with their corresponding MFRs as well as short description of chemical structures in Table 1.

The six compatibilizer candidates used for the model HDPE rich HDPE/PP blend are an ethylene-based olefin block copolymer (OBC) (C1), a propylene-based olefin block copolymer (OBC) (C2), two propylene-based ethylene propylene random copolymers (EPR) (C3 and C4) and two styrene-ethylene-butylene-styrene triblock copolymers (SEBS) (C5 and C6). The propylene-based ethylene propylene random copolymers differ from each other in their ethylene contents as well as MFRs. Furthermore, one of the styrene-ethylene-butylene-styrene triblock copolymers (C5) has 13% polystyrene content, whereas another styrene-ethylene-butylene-styrene triblock copolymer (C6) has 20% polystyrene content.

### 2.2. Rheological Characterization of Materials

The samples with 25 mm diameter as well as 1.2 mm thickness were cut out from compression molded sheets for dynamic rheology measurements. The rheological analysis of the samples was carried out in parallel-plate-mode using Anton Paar MCR 301 rheometer equipped with CTD 450 heating chamber under nitrogen at 240 °C with 1 mm gap size. The deformation raised logarithmically from 1% to 2% with a frequency sweep from 628 to 0.01 rad/s during measurements.

**Figure 1:**
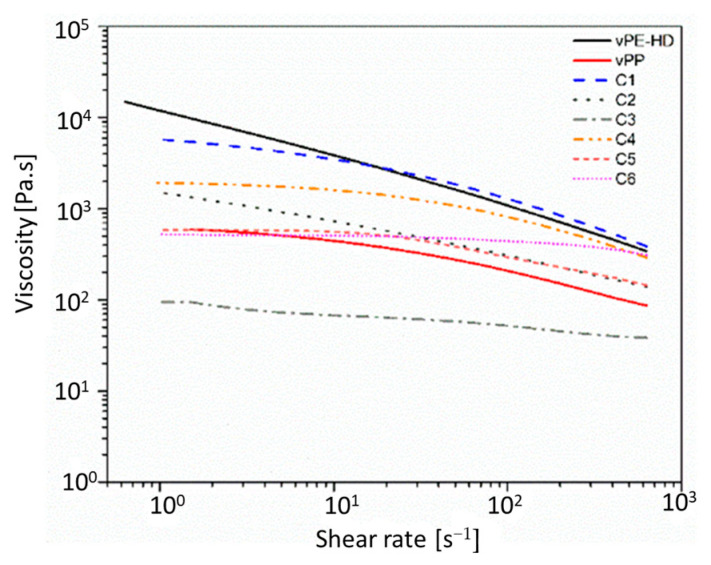
Melt viscosity of used materials at 240 °C.

The measured shear rate dependence of melt viscosity curves of materials is provided in Figure 1.

It is worth underling that the high molecular weight of HDPE has a similar viscosity to that of ethylene-based olefin block copolymer (C1) between 10^1^–10^3^ shear range at 240 °C. Furthermore, the ethylene propylene random copolymer (C4) with a melt viscosity of 1000 Pa.s has the lowest melt viscosity at 240 °C.

### 2.3. Sample Preparation

The vHDPE and vPP were first ground into smaller particles with a diameter of 3 mm in a Fritsch granulator “Pulverisette 16”. The vHDPE, vPP, blend including 90% vHDPE and 10% vPP without compatibilizers, and the blends with 5% wight amount of six different kinds of compatibilizer candidates were separately extruded at 240 °C in Haake Minilab 2 twin screw extruder with 30 s residence time and 100 rpm screw speed. The generated strands from extrusion were cut into small pieces and then compression molded into sheets using the Collin P 200 P heating press at 190 °C. The following parameters were adjusted for compression molding: preheating at 150 °C under 8 bar pressure for 10 min, heating from 150 °C to 190 °C at 22 bar for 8 min, compressed by the melting temperature noted above (190 °C) at 30 bar for 5 min and then cooled with 10 K/min from 190 °C to 30 °C at 35 bar for 20 min. The sample specifications and abbreviation are summarized in Table 2.

### 2.4. Contact Angle Measurements

The contact angle measurements were performed by using contact angle meter (Krüss DSA 30). The static contact angle of used materials (PP, HDPE and compatibilizer candidates) were determined relative to water (H_2_O) and diiodomethane (CH_2_I_2_) using the sessile drop (3 µL) methods. The used materials, as shown in Table 2, were compression molded at 190 °C prior to contact angle measurements. The surface tension of HDPE, compatibilizers, PP were calculated using Wu’s equation [28]:

(1) $$\left(1+cos\theta_{H_{2}O}\right)\gamma_{H_{2}O}=4\left(\frac{\gamma_{H_{2}O}^{d}\gamma^{d}}{\gamma_{H_{2}O}^{d}+\gamma^{d}}+\frac{\gamma_{H_{2}O}^{p}\gamma^{p}}{\gamma_{H_{2}O}^{p}+\gamma^{p}}\right)$$

(2) $$\left(1+cos\theta_{CH_{2}I_{2}}\right)\gamma_{CH_{2}I_{2}}=4\left(\frac{\gamma_{CH_{2}I_{2}}^{d}\gamma^{d}}{\gamma_{CH_{2}I_{2}}^{d}+\gamma^{d}}+\frac{\gamma_{CH_{2}I_{2}}^{p}\gamma^{p}}{\gamma_{CH_{2}I_{2}}^{p}+\gamma^{p}}\right)$$

(3) $$\gamma_{A,B,C}=\gamma_{A,B,C}^{d}+\gamma_{A,B,C}^{p}$$

where *θ* is the contact angle, *γ* is the surface energy (mN m^−1^). The polar component and the dispersed component are *γ^p^* and *γ^d^*, respectively. The indices *A*, *B* and *C* symbolize the components in ternary blends. In this case, the ternary blend components are HDPE, PP and compatibilizer candidates.

### 2.5. Morphological Analysis

The morphologies of blends were analyzed using a Philips Model XL30 scanning electron microscopy (SEM). The samples were first cut at −120 °C using rotation microtom (Mikrom HM360). Consequently, the fractured surfaces were etched with n-heptane at 80 °C for 5 h and then sputtered with gold layer.

### 2.6. Adhesion Tests

**Figure 2:**
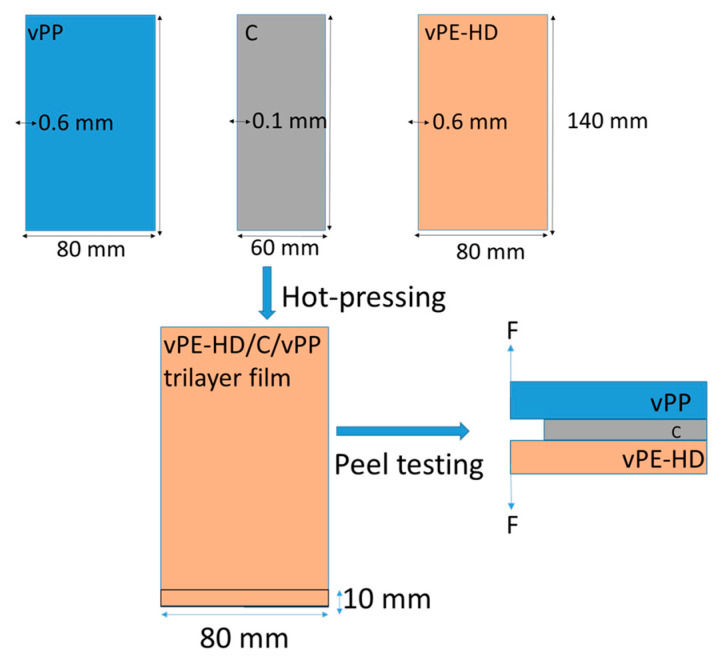
Schematic representation of sample geometry and sample preparation.

The adhesion of compatibilizer candidates to HDPE and PP were measured using peel testing. The sheets of HDPE with geometry of 140 mm × 80 mm × 0.6 mm, PP with geometry of 140 mm × 80 mm × 0.6 mm and compatibilizers with geometry of 140 mm × 60 mm × 0.1 mm were hot pressed at 190 °C by using a mold with a geometry of 140 mm × 80 mm, then sandwiched together (HDPE/Compatibilizers(C)/PP) and consequently once again hot pressed at 240 °C 30 s to simulate extrusion process. The hot press machine used was a Collin P 200 P heating press. The schematic representation of samples was shown in Figure 2.

For the peel tests, the sandwiched sheets by compression molding were cut with a sample width of 10 mm. The obtained sandwiched sheets were placed in tensile tester (Zwick 050) equipped with a 1 kN load cell. The samples were pulled apart at 10 mm/min. The peel force was measured as a function of displacement. The peel strength (N/mm) was displayed as the peel force divided by the sample width.

### 2.7. Mechanical Properties

For the tensile test specimens, approximately 22 g of small pieces from extruded strands were cut, weighted, and pressed into sheets with 1.8–1.9 mm thickness at 190 °C. The molded sheets were then punched out into tensile test specimens according to ISO 572-2 type 5A. Seven tensile test specimens were punched out for tensile test measurements to ensure reproducibility. Tensile tests were conducted at 23 °C using a test machine (Zwick 050) equipped with a 1 kN load cell and an extensometer with 10 mm/min test speed.

For the tensile impact test specimens, approximately 12 g of small pieces from extruder strands were cut, weighted and pressed into sheets with 1.1–1.2 mm thickness at 190 °C. These sheets were then punched out into tensile impact test specimens according to the ISO 8256 method A. Afterwards, the test specimens were notched using a Notch-Vis (Ceast, Darmstadt, Germany) on both sides. The tensile impact tests were performed by an Instron CAEST 9050 (Ceast, Darmstadt, Germany) impact pendulum equipped with 2 J hammer and 15 g of a cross head mass. For the reproducibility, seven tensile impact test specimens were measured.

## 3. Results and Discussion

### 3.1. Prediction of Morphology by Contact Angle Measurements

To predict the morphology of ternary blends, the contact angles of vHDPE, vPP, and compatibilizer candidates were measured. According to Equations (1)–(3), the polar component (p) and dispersed component (d) of surface tension were calculated. The obtained results are shown in Table 3.

However, the surface tension strongly depends on temperature as well as molecular weight of polymer [29]. Therefore, the surface tension of ternary blend components should be calculated at processing temperature of melt blending. The process temperature of melt blending is 240 °C. To estimate the morphology of different ternary blends, the surface tension of ternary blend components at 240 °C can be determined using the empirical Equation (4) by Guggenheim [30]:

(4) $$\gamma_{S,T_{m}}=\gamma_{S,T_{0}}{\left(1-\frac{T_{0}}{T_{m}}\right)}^{11/9}$$

where $\gamma_{S,T_{m}}$ and $\gamma_{S,T_{0}}$ represent the surface tension at blending temperature ($T_{m}$) and testing temperature ($T_{0}),$ respectively. The calculated surface tension according to Equation (4) is provided in Table 4.

After the calculation of surface tension of ternary blend components at process temperature (240 °C), the interfacial tension of possible component pairs, such as *γ_AB_*, *γ_AC_* and *γ_BC_*, were calculated using the well-known harmonic mean Equation [31]:

(5) $$\gamma_{AB}=\gamma_{A}+\gamma_{B}-4\left(\frac{\gamma_{A}^{d}\gamma_{B}^{d}}{\gamma_{A}^{d}+\gamma_{B}^{d}}+\frac{\gamma_{A}^{p}\gamma_{B}^{p}}{\gamma_{A}^{p}+\gamma_{B}^{p}}\right)$$

Furthermore, the adhesive energy can be calculated using Equation (6).

(6) $$W_{AB}=2{\left(\gamma_{A}^{d}\gamma_{B}^{d}\right)}^{1/2}2{\left(\gamma_{A}^{p}\gamma_{B}^{p}\right)}^{1/2}$$

The calculated interfacial tension as well as adhesive energy (*W_AB_*) at 240 °C are summarized in Table 5.

Comparing all results, one can see that only the ethylene-based olefin block copolymer (C1) as a ternary blend component leads to a decrease in interfacial tension from 3.67 mN/m to 2.29 mN/m and an increase in adhesive energy from 61.86 mN/m to 64.75 mN/m. As can be seen in Table 5, the adhesive energy of vHDPE/C1 (64.75 mN/m) is higher than vHDPE/vPP (61.86 mN/m). The result suggests that vHDPE has a better compatibility with ethylene-based olefin block copolymer (C1). According to the literature, such decrease in interfacial tension as well as the increase in adhesive energy during the melt blending and extrusion process is evidence of good adhesion as well as core-shell formation, in which the dispersed phase is encapsulated by compatibilizer [32,33,34]. In this case, dispersed vPP in vHDPE matrix is encapsulated only by the ethylene-based olefin block copolymer (C1).

The interfacial tension plays an important role in ternary blend morphology. The Hobbs spreading coefficient theory describes the relationship between interfacial tension and ternary blend morphology [35]. To predict ternary blend morphology by the interfacial tensions between different blend component pairs, the Hobbs spreading coefficient theory is mostly used [11,12,13,14]. The spreading coefficient (*λ*) can be calculated using Equations (7)–(9):

(7) $$\lambda_{BC}=\gamma_{AC}-\left(\gamma_{AB}+\gamma_{BC}\right)$$

(8) $$\lambda_{CB}=\gamma_{AB}-\left(\gamma_{AC}+\gamma_{BC}\right)$$

(9) $$\lambda_{AB}=\lambda_{AC}=\gamma_{BC}-\left(\gamma_{AB}+\gamma_{AC}\right)$$

**Figure 3:**
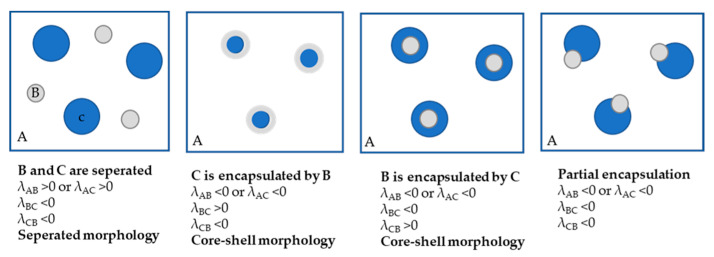
Schematic representation of common possible phase morphologies in ternary blends (*A*/*B*/*C*) [36].

In the above equations, the *λ_AB_*, *λ_BC_* and *λ_CB_* represent the spreading coefficients *A* over *B*, *B* over *C* and *C* over *B*, respectively. Based on the spreading coefficients, the phase morphology of ternary blends can be estimated. For the estimation of ternary blend morphology, a dispersed phase diagram (Figure 3) is generally used.

To predict the morphology of six ternary blends, the spreading coefficients are calculated according to Equations (5)–(7). The spreading coefficients of component couples of six ternary blends with corresponding morphologies are determined according to dispersed phase diagram (Figure 3) and shown in Table 6.

Table 6 represents the spreading coefficients and predictions of phase morphology of vHDPE/vPP ternary blends with six compatibilizer candidates.

**Figure 4:**
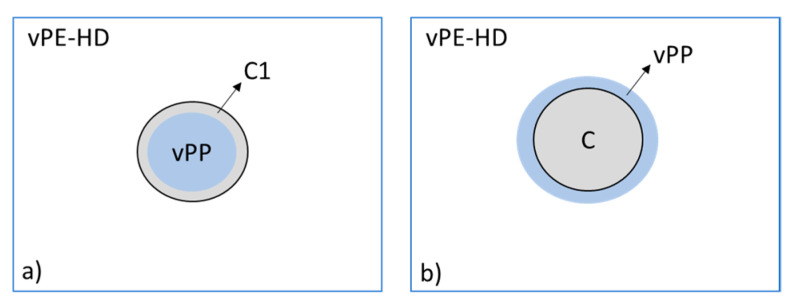
Schematic representation of estimated morphology according to spreading coefficient theory, (**a**) the morphology of vHDPE/C1/vPP ternary blend, (**b**) the morphology of vHDPE/C2, C3, C4, C5 and C6/vPP ternary blends.

According to the spreading coefficient theory, the ternary blend of vHDPE/vPP with ethylene-based olefin block copolymer as a compatibilizer (C1) has a unique core-shell morphology due to λ_C1/vPP_ > 0, λ_vPP/C1_ < 0 and λ_vHDPE/C1_ = λ_vHDPE/vPP_ < 0. This result indicates that the dispersed vPP particle in vHDPE matrix is encapsulated by the ethylene-based olefin block copolymer (C1) as a compatibilizer, as schematically illustrated in Figure 4a.

Conversely, the ternary blend of vHDPE/vPP with other compatibilizer candidates, such as propylene-based olefin block copolymer (C2), ethylene-propylene random copolymers (C3 and C4) and styrene-ethylene-butylene-styrene triblock copolymers (C5 and C6), is encapsulated by vPP according to spreading coefficient theory due to the λ_C1/vPP_ < 0, λ_vPP/C1_ > 0 and λ_vHDPE/C1_ = λ_vHDPE/vPP_ < 0, as illustrated in Figure 4.

The encapsulation of PE in PP matrix by propylene-based olefin block copolymer [26,32,33], ethylene propylene random copolymers (EPR) [13,37] and styrene-ethylen-butylene-stryrene triblock copolymers (SEBS) [27] has been reported in the literature. However, the encapsulation of vPP in vHDPE matrix by propylene-based olefin block copolymer (C2) or ethylene-propylene-random copolymers (C3 and C4) or styrene-ethylene-butylene-styrene triblock copolymers (C5 and C6) has not been found in this study. It must be underlined that the encapsulation of PP in PE matrix by compatibilizer (core-shell morphology) depends on the interfacial tension between compatibilizer and PP or PE. The interfacial tension is related to viscosity and molecular weight of ternary blend components [29]. In our study, the blow molding grade of HDPE with high molecular weight as a matrix and the injection molding grade PP with significantly low molecular weight as a dispersed phase were used, whereas PP as a matrix and PE as a dispersed phase with not huge difference in average molecular weight were used in the above-mentioned studies. In other words, it cannot be generalized that the used compatibilizer always encapsulates the dispersed phase because the morphology of ternary blend depends on interfacial tension, which is related with the average molecular weight of the ternary blend component.

Consequently, the ethylene-based olefin block copolymer (C1) can only encapsulate the dispersed vPP phase in vHDPE matrix to form core-shell morphology, whereas the other compatibilizer candidates are encapsulated by dispersed vPP in vHDPE matrix. The predicted two different morphologies (Figure 4a,b) in ternary blends are considered for interpretation of mechanical performance in the next chapter.

This methodology can be used for morphology prediction of compatibilized recycled rHDPE-rich-rHDPE/rPP blends from post-consumer detergent bottle waste. However, it should be noticed that the detergent residues in recycled bottle waste could affect the results of contact angle measurements and thereof the predicted morphology.

### 3.2. Morphological Analysis

**Figure 5:**
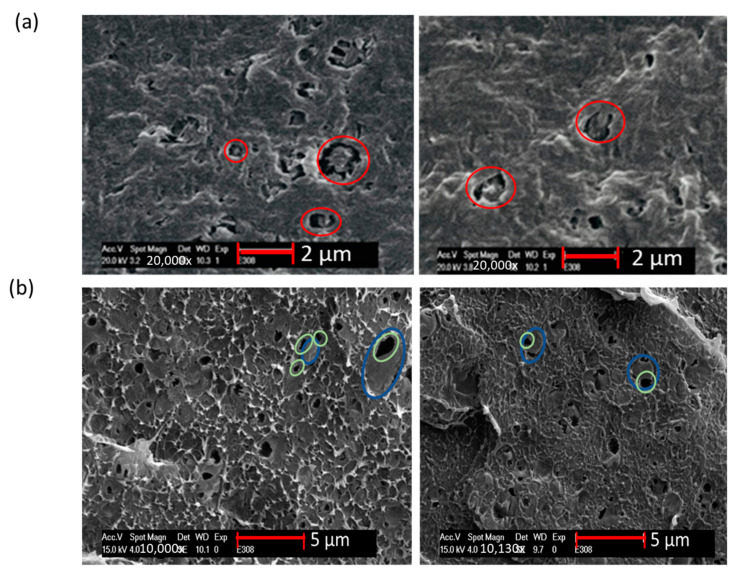
SEM images of 10 wt% vPP contaminated vHDPE/vPP blend with (**a**) 5 wt% of ethylene-based olefin block copolymer (C1) and (**b**) 5 wt% of ethylene-propylene-random copolymer (C3).

Figure 5a shows the SEM images of 10 wt% vPP contaminated vHDPE/vPP blend with 5 wt% of ethylene-based olefin block copolymer (C1) as a compatibilizer. The ethylene-based olefin block copolymer was etched in the matrix around the dispersed PP phase, as seen in Figure 5a within red circles. This confirms the core-shell morphology of the ternary blend with ethylene-based olefin block copolymer (C1), as predicted using spreading coefficients in the previous section and shown in Figure 4a.

The SEM images of 10 wt% vPP contaminated vHDPE/vPP blend with 5 wt% of ethylene-propylene random copolymer (C3) are shown in Figure 5b. One can see that the ethylene-propylene-random copolymer was etched, as seen on the dark particles marked with green circles. The etched ethylene-propylene-random copolymer particles are located in dispersed PP particles (blue circles), which confirms the predicted morphology in Figure 4b.

### 3.3. Adhesion Tests

**Figure 6:**
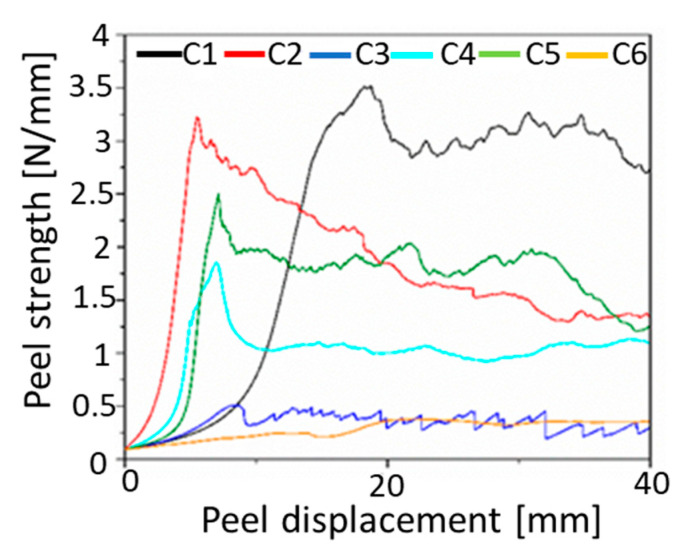
The results of adhesion tests for six vHDPE/compatibilizer/vPP trilayer films.

The adhesion of compatibilizer candidates to vHDPE as well as vPP is an important factor that determines the mechanical performance of compatibilized blends by means of their contribution to the enhancement of stress transfer between phases. The adhesion is correlated with peel strength. The peel strengths of six trilayer films (vHDPE/C/vPP) with six compatibilizer candidates as tie layers are shown in Figure 6.

As seen in Figure 6, the best adhesion on vHDPE as well as vPP is achieved by using ethylene-based olefin block copolymer (C1) as an interfacial layer in vHDPE/vPP laminate due to higher peel strength of C1 compared to other compatibilizers. As already reported, the better adhesion of ethylene-based olefin block copolymer to polyethylene and polypropylene is originated from the miscibility of soft octene segment with polypropylene as well as the miscibility of hard ethylene segment with polyethylene [38]. The miscibility of ethylene-based olefin block copolymer with both components results in combining two polymers with interfacial entanglements.

Furthermore, the ethylene-propylene random copolymer (C3) and styrene-ethylene-butylene-polystyrene triblock copolymer (C6) have the lowest peel strengths, whereas the propylene-based olefin block copolymer (C2), ethylene-propylene random copolymer (C3) and styrene-butylene-polystyrene triblock copolymer (C5) have peel strengths in between. The significant difference between compatibilizer candidates are viscosity curves (Figure 1) and therefore molecular weights. The higher molecular weight due to the low MFR (0.6 g/10 min @ 190 °C, 2.16 kg) as well as high viscosity (Figure 1) is the ethylene-based olefin block copolymer (C1), which has a superior adhesion to blow molding grade of vHDPE and injection molding grade of vPP.

According to the literature, the copolymers with insufficient molecular weights have the worse adhesion due their low interfacial entanglements [16]. Conversely, the block copolymers with high molecular weight have the best adhesion due to the higher degree of entanglements, as reported in the literature [15]. Therefore, the best adhesion of ethylene-based olefin block copolymer (C1) to vHDPE and vPP can be explained by the higher molecular weight of ethylene-based olefin block copolymer (C1) as well as the above-mentioned interfacial interaction. The higher molecular weight of C1 enables more interfacial entanglements at the interface between vHDPE and vPP, which is confirmed also in other studies [23,24,39]. As a result, the ethylene-based olefin copolymer (C1) not only encapsulates the dispersed vPP phase in the vHDPE matrix (core-shell morphology) but also has better adhesion to vHDPE and vPP due to the high molecular weight as well as interfacial interaction with vHDPE and vPP. Due to the better adhesion of ethylene-based olefin block copolymer (C1) to virgin blow molding grade of HDPE as well as injection molding grade of PP, the ethylene-based olefin block copolymer (C1) can be used as a compatibilizer for recycled post-consumer detergent bottle waste, which consist of blow molding grade rHDPE and injection molding grade of rPP. However, it should be considered that the recycled rHDPE (blow molding grade)-rich rHDPE (blow molding grade)/rPP (injection molding grade) blend from post-consumer detergent bottles contains pigments as well as detergent residues. These additional contaminants can reduce adhesion of ethylene-based olefin block copolymer (C1) to blow molding grade rHDPE and injection molding grade rPP.

### 3.4. Mechanical Properties

**Figure 7:**
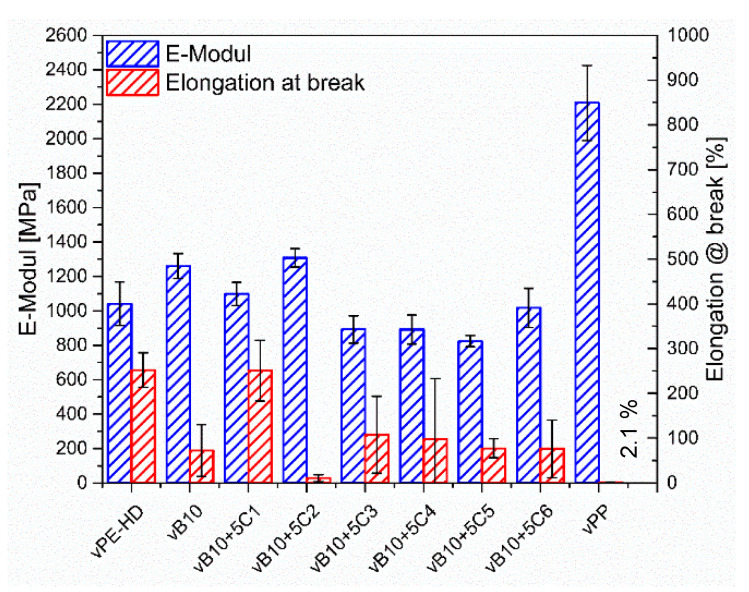
Tensile test results of vHDPE, vPP, vB10 and vB10 with 5 wt% of six different compatibilizer candidates.

The effects of adding 5 wt% of various compatibilizer candidates to 10 wt% vPP contaminated vHDPE (vB10 model blend) on tensile properties were investigated. The tensile test results of vHDPE, vPP, vB10 and vB10 with 5 wt% of six different compatibilizer candidates (C1, C2, C3, C4, C5 and C6) are provided in Figure 7.

The compression molded vHDPE homopolymer demonstrates 600% of elongation at break, whereas the compression molded vPP homopolymer shows the lowest elongation at break at about 2.1%, the highest E-Modulus as well as tensile strength at about 2200 MPa and 25 MPa, respectively. The compression molded vPP (injection molding grade) is brittle because of the high MFR. The addition of 10 wt% of vPP to vHDPE (vB10) results in significant reduction of the elongation at break from 600% to 200%. The reduction in elongation at break is approximately 67%. The brittle behavior of vB10 is due to the immiscibility between vHDPE and vPP homopolymers. The immiscibility between vHDPE and vPP leads to phase separated morphology. According to the literature, the reasons for deterioration in elongation at break of blends are immiscibility, phase separated morphology, insufficient number of interfacial entanglements at the interface between blend components and therefore high surface tension as well as weak adhesion [10]. The weak adhesion between matrix and dispersed phase causes poor stress transfer between phases and consequently poor mechanical performance. The reduction of interfacial tension, enhancement of adhesion between phases and improvement of mechanical performance can be achieved by the addition of a proper additive, known as a compatibilizer, which is miscible with polymer blend components and migrates through the interface boundary to improve stress transfer between phases by interfacial entanglements [16,20,40,41].

**Figure 8:**
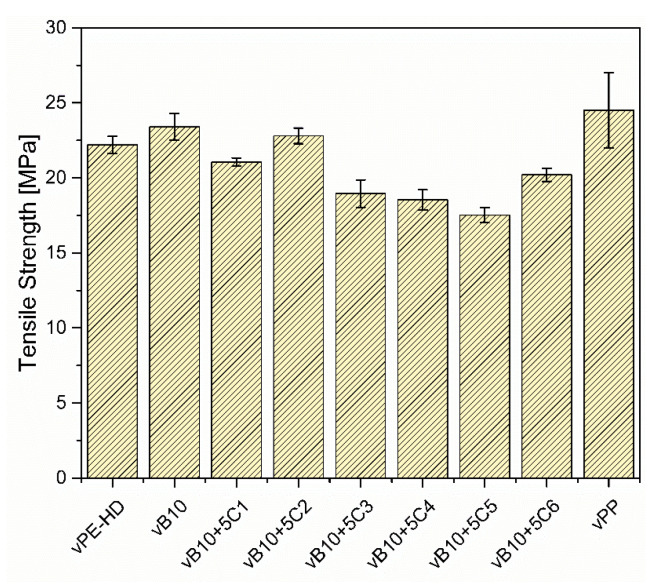
Tensile strength results of vHDPE, vPP, vB10 and vB10 blended with 5 wt% of six different compatibilizer candidates.

Figure 7 and Figure 8 illustrate the comparison with tensile properties of six different types of compatibilizer candidates for 10 w-% vPP contaminated vHDPE/vPP blend (vB10). On the one hand, the addition of 5 wt% of ethylene-based olefin block copolymer (C1) to 10 wt% vPP contaminated vHDPE (vB10) enhances the elongation at break from 200% to 600%, which is approximately the same elongation at break of vHDPE homopolymer. In other words, the deterioration of elongation at break for 10 w-% vPP contaminated vHDPE/vPP blend (vB10) is repaired by the addition of 5 wt% of ethylene-based olefin block copolymer (C1). The significant improvement in elongation at break with the addition of 5 wt% ethylene-based olefin block copolymer as a compatibilizer (C1) in vB10 can be attributed to the fact that the dispersed vPP is encapsulated by ethylene-based olefin block copolymer (C1), forming a core-shell morphology in the vHDPE matrix, as estimated by the spreading coefficient (Figure 4a). This core-shell morphology (Figure 4a) as well as better adhesion of ethylene-based olefin block copolymer (C1) to vHDPE and vPP, as seen in Figure 6, enables a significant improvement in stress transfer between phases and consequently in elongation at break. The increase in elongation at break of PE/PP blend with the addition of olefin block copolymer (OBC) as a compatibilizer owing to the core-shell morphology and better adhesion of olefin block copolymer to blend components has been reported in the literature [24,26,32,38,42], which confirms the obtained results. On the other hand, the addition of 5 wt% of propylene-based olefin block copolymer (C2), ethylene-propylene random copolymers (C3 and C4) and styrene-ethylene-butylene-styrene triblock copolymers (C5 and C6) to 10 wt% vPP contaminated vHDPE (vB10) leads to no remarkable improvement in elongation at break and instead to even more deterioration in elongation at break. The observed deterioration of elongation at break is related to blend morphology and adhesion of compatibilizers to vPP and vHDPE. The encapsulation of some compatibilizers (C2, C3, C4, C5 and C6) by vPP in the vHDPE matrix (Figure 4b) and the insufficient adhesion of these compatibilizers to vPP and vHDPE lead to poor elongation at break due to the poor stress transfer between phases during applied load in uniaxial direction.

The E-Modulus and tensile strength of vHDPE are also influenced by the addition of compatibilizers, as seen in Figure 7 and Figure 8. It can be observed in Figure 7 and Figure 8 that the addition of 10 wt% of vPP into vHDPE (vB10) results in increasing E-Modulus and tensile strength due to the high E-Modulus as well as tensile strength of vPP. The E-Modulus and tensile strength slightly increase with the addition of propylene-based olefin block copolymer (C2) to vB10, whereas the addition of ethylene-based olefin block copolymer (C1), ethylene propylene random copolymers (C3 and C4) and styrene-ethylene-butylene-styrene triblock copolymers (C5 and C6) to vB10 leads to a significant decrease in E-Modulus as well as tensile strength. The significant reduction in E-Modulus and tensile strength is caused by low E-Modulus as well as tensile strength of compatibilizers, which lead to a decrease in the overall E-Modulus and tensile strength of blends, as reported in the literature [42,43,44,45,46]. As a result, the ethylene-based olefin block copolymer (C1) is the proper compatibilizer for vB10 with remarkable improvement in elongation at break and without any noticeable loss in E-Modulus and tensile strength compared to that of neat vHDPE.

**Figure 9:**
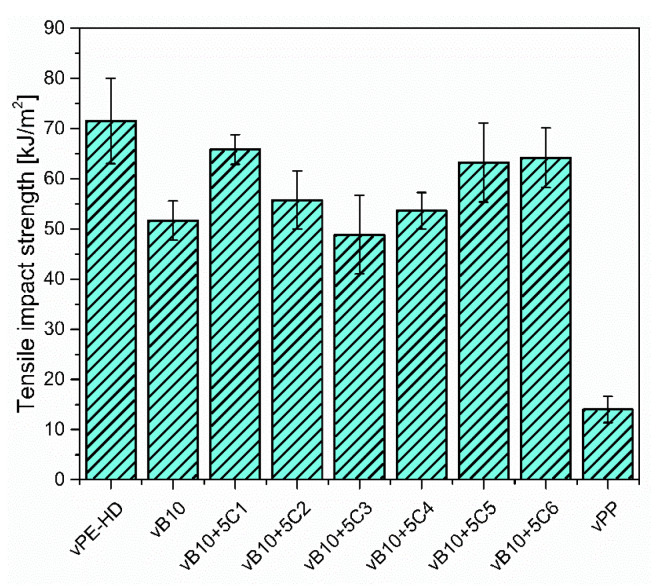
Tensile impact strength results of vHDPE, vPP, vB10 and vB10 blended with 5 wt% of six different compatibilizer candidates.

In addition to the tensile testing, the tensile impact testing provides important information regarding impact resistance under dynamic load. The tensile impact strength results of vHDPE, vPP, vB10 and vB10 blended with 5 wt% of different compatibilizers are shown in Figure 9.

Here, one can see that the compression molded vPP (injection molding grade) shows brittle behavior with a tensile impact strength of 15 kJ/m^2^ due to the high MFR, low viscosity and low molecular weight of vPP. The addition of 10 wt% of vPP to vHDPE deteriorates the tensile impact strength of vHDPE from 72 kJ/m^2^ to 52 kJ/m^2^. The reduction in tensile impact strength is of approximately 30%. The deterioration in impact strength with the addition of PP to PE or PE to PP has been reported in the literature [47,48,49,50]. They found that the immiscibility, the lack of adhesion between phases and the high interfacial tension led to a worsening of the impact strength of polymer blends. The obtained results are fully in accordance with the above-mentioned literature. It can be seen in Figure 9 that the 5 wt% addition of ethylene-based olefin block copolymer (C1) or styrene-ethylene-butylene-styrene triblock copolymers (C5 and C6) to 10 wt% vPP contaminated vHDPE (B10) results in an increase of tensile impact strength for B10 blend, which is more pronounced by ethylene-based olefin block copolymer (C1). The target tensile impact strength of neat vHDPE can be achieved by the addition of ethylene-based olefin block copolymer (C1) as well as styrene-ethylene-butylene- styrene triblock copolymers (C5 and C6), as seen in Figure 9. It is worth underlining that the encapsulation of dispersed vPP by ethylene-based olefin block copolymer (C1) in vHDPE matrix (core-shell morphology) for vB10 blend causes resistance to crack propagation and therefore a significant increase in tensile impact strength compared to what happens without compatibilizer. Furthermore, the improvement in tensile impact strength of vB10 with the addition of styrene-ethylene-butylene-styrene triblock copolymers (C5 and C6) is related to the impact modifier effect by the addition of thermoplastic elastomers, which are encapsulated by vPP in the vHDPE matrix, as estimated according to the spreading coefficient and illustrated in Figure 4b. On the other hand, the target tensile impact strength of vHDPE cannot be achieved with the addition of 5 wt% of propylene-based olefin block copolymer (C2) or ethylene propylene random copolymers (C3 and C4) to vB10 blend. Even worse tensile impact strengths are observed with the addition of ethylene-propylene random copolymers (C3 and C4) due to the poor adhesion of these copolymers to vPP and vHDPE, as shown in Figure 6. Consequently, the ethylene-based olefin block copolymer (C1) is the most suitable compatibilizer for the compatibilization of 10 wt% vPP contaminated vHDPE blend (vB10) due to the high tensile impact strength originated from high peel strength (Figure 6) as well as the encapsulation of dispersed vPP particles by ethylene-based olefin block copolymer in vHDPE matrix (core-shell morphology). The enhancement in impact strength for PP-rich PP/PE blends with the addition of propylene-based olefin block copolymer has been also reported in the literature [24,26,32,38,42]. According to these studies, the improvement in impact strengths of PP-rich PP/PE blends are related to the encapsulation of dispersed PE phase by propylene-based olefin block copolymer in PP matrix and superior adhesion to polymer blend components due to the interfacial entanglements via miscible segments of propylene-based olefin block copolymer with PP as well as PE, as mentioned above in the adhesion section. Conversely, in this study, the tensile impact strength of PE-rich PE/PP blend is improved with the addition of ethylene-based olefin block copolymer (C1), which is also confirmed by the results obtained from the compatibilization of PP-rich PP/PE blends with the addition of propylene-based olefin block copolymer in the above-mentioned studies.

**Figure 10:**
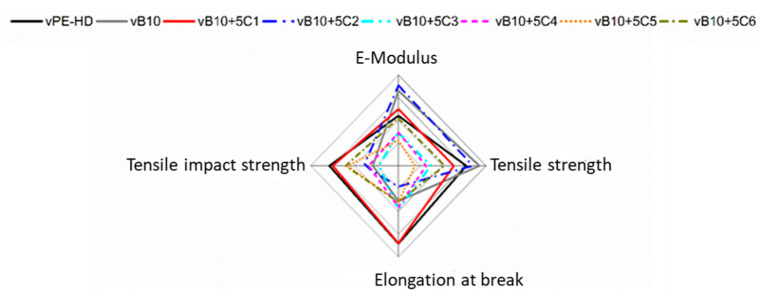
Overall mechanical performance of vHDPE, vB10 and vB10 with 5 wt% of various types of compatibilizer candidates.

The increasing in elongation at break as well as impact strength is a significant evident for enhancement of phase adhesion and successful compatibilization, as reported in the literature [19,41,50,51,52]. Consequently, the successful compatibilization of 10 wt% vPP contaminated vHDPE (vB10 blend) is only achieved with 5 wt% addition of the ethylene-based olefin block copolymer (C1) due to the improvement in elongation at break and tensile impact strength. In other words, the modification of 10 wt% vPP (injection molding grade) contaminated vHDPE (blow molding grade) blend (vB10) with 5 wt% of ethylene-based olefin block copolymer (C1) yielded a material with mechanical properties very close to neat vHDPE, as illustrated in radar charts in Figure 10. The obtained results from tensile as well as tensile impact tests virgin model blends highlighted that ethylene-based olefin block copolymer (C1) can be used as a compatibilizer in order to improve impact resistance of rHDPE-rich-rHDPE/rPP blend from recycled post-consumer detergent bottle waste.

## 4. Conclusions

Selection of an effective compatibilizer within the industrially available compatibilizers for the recycling of PE-rich PE/PP blends can be challenging. This study investigated the compatibilization of vHDPE contaminated with 10 wt.% vPP and proposes a new methodology for rapidly identifying optimal compatibilizers based on basic surface properties, such as static contact angles, spreading coefficients, adhesion strength, tensile and tensile impact properties and surface morphology. The proposed methodology comprises two distinct stages, initially, prediction of ternary blend morphology using contact angle derived spreading coefficients, and secondly, evaluation of the adhesion strength of the compatibilizer to ternary blend components. The morphology of six ternary blends modelled using their spreading coefficients revealed that only ethylene-based olefin block copolymer (C1) exhibited core-shell morphology, with C1 as a shell, vPP as a core and a vHDPE matrix. This core-shell morphology is critical for successful compatibilization. The other investigated compatibilizers were encapsulated by vPP in HDPE matrix. C1 also provided the best adhesion strength of the candidates due to its high viscosity, which matched vHDPE in processing temperature and shear rate. PE/PP blends compatibilized with as little as 5 wt.% C1 exhibited 67% higher elongation at break and increases in tensile impact of 25%. Future research should further investigate the role of compatibilizer adhesion and core-shell morphology in the mechanical properties of blends. The identification of C1 as an optimal compatibilizer supports its widespread adoption for improving PP contaminated HDPE recycled material into virgin grade material, a move which could improve recycling yields to meet increasingly ambitious EU targets by allowing the use of more deteriorated post-consumer waste as feedstock and improving consumer confidence in the quality and properties of recycled materials. The proposed methodology can also be widely used for the screening and accelerated identification of optimal compatibilizers for other polymer blends and recycling streams, which will be absolutely critical in the revolution and expansion of the recycling sector as it moves to become the central player in the circular economy of the future.

## Figures and Tables

**Table 1:** Materials used in the study.

Materials	MFR (g/10 min)	Description
vHDPE	0.4 ^a^	Virgin HDPE (Lyondellbasell)
vPP	20 ^b^	Virgin PP (Borealis)
C1	0.5 ^a^	Ethylene-based OBC (Dow Chemical Company)
C2	6.5 ^b^	Propylene-based OBC (Dow Chemical Company)
C3	20 ^a^	Propylene-based EPR (DuPont)
C4	1.4 ^a^	Propylene-based EPR (ExxonMobil Chemical)
C5	19 ^b^	SEBS (Kraton)
C6	22 ^c^	SEBS (Kraton)

^a^ 190 °C, 2.16 kg, ^b^ 230 °C, 2.16 kg, ^c^ 230 °C, 5 kg.

**Table 2:** Samples specification and abbreviation.

Sample Specification	Abbreviation
Virgin blow molding grade of HDPE	vHDPE
Virgin injection molding grade of PP	vPP
vHDPE with 10 wt% vPP contamination	vB10
vHDPE with 10 wt% vPP contamination + 5 wt% C1	vB10 + 5C1
vHDPE with 10 wt% vPP contamination + 5 wt% C2	vB10 + 5C2
vHDPE with 10 wt% vPP contamination + 5 wt% C3	vB10 + 5C3
vHDPE with 10 wt% vPP contamination + 5 wt% C4	vB10 + 5C4
vHDPE with 10 wt% vPP contamination + 5 wt% C5	vB10 + 5C5
vHDPE with 10 wt% vPP contamination + 5 wt% C6	vB10 + 5C6

**Table 3:** Contact angles and surface tensions of vHDPE, vPP and compatibilizer candidates at 25 °C.

Sample	θH_2_O (°)	θCH_2_H_2_ (°)	γ (mN/m)	γ^d^ (mN/m)	γ^p^ (mN/m)
vHDPE	78.4	54.8	40.43	28.87	11.56
vPP	97.6	57.3	32.59	29	3.59
C1	111.8	52.8	35	30	5
C2	102.2	51.4	34.79	33.65	1.14
C3	102.2	66.9	27.67	24.75	2.92
C4	105	68.8	26.42	24.34	2.07
C5	96.5	52.1	35.04	31.59	3.45
C6	108.1	64.5	28.81	28.64	0.17

**Table 4:** Calculated surface tension for vHDPE, vPP and compatibilizers at 240 °C.

Sample	γ (mN/m)	γ^d^ (mN/m)	γ^p^ (mN/m)
vHDPE	35.35	25.24	10.11
vPP	28.49	25.35	3.14
C1	30.6	26.23	4.37
C2	30.41	29.41	1
C3	24.19	21.64	2.55
C4	23.09	21.28	1.81
C5	30.63	27.61	3.02
C6	25.19	25.04	0.15

**Table 5:** Interfacial tensions between various components of ternary blends at 240 °C.

Ternary Blend	Component Pair	Interfacial Tension (mN/m)	Adhesive Energy (mN/m)
vHDPE/C1/vPP	vHDPE/vPP	3.67	61.86
	vHDPE/C1	2.29	64.75
	vPP/C1	0.22	58.98
vHDPE/C2/vPP	vHDPE/vPP	3.67	61.86
	vHDPE/C2	7.79	60.86
	vPP/C2	1.41	58.1
vHDPE/C3/vPP	vHDPE/vPP	3.67	61.86
	vHDPE/C3	4.79	56.89
	vPP/C3	0.35	52.46
vHDPE/C4/vPP	vHDPE/vPP	3.67	61.86
	vHDPE/C4	6.12	54.9
	vPP/C4	0.71	51.21
vHDPE/C5/vPP	vHDPE/vPP	3.67	61.86
	vHDPE/C5	3.93	63.8
	vPP/C5	0.1	59.06
vHDPE/C6/vPP	vHDPE/vPP	3.67	61.86
	vHDPE/C6	9.67	52.74
	vPP/C6	2.72	51.76

**Table 6:** Spreading coefficients between various ternary blend components at 240 °C.

Ternary Blend (*A*/*B*/*C*)	*λ_BC_*	*λ_CB_*	*λ_AB_* = *λ_AC_*	Morphology Prediction
vHDPE/C1/vPP	1.16	−1.6	−5.74	Core-shell morphology
vHDPE/C2/vPP	−5.53	2.71	−10	Core-shell morphology
vHDPE/C3/vPP	−1.5	0.77	−8.1	Core-shell morphology
vHDPE/C4/vPP	−3.16	1.73	−9.1	Core-shell morphology
vHDPE/C5/vPP	−0.4	0.17	−7.5	Core-shell morphology
vHDPE/C6/vPP	−8.7	3.3	−10.6	Core-shell morphology

## Data Availability

The data presented in this study are available on request from the corresponding author.
